# Clinical and echocardiographic outcomes of sync-atrioventricular versus nominal optimization in correlation with QRS narrowing among CRT patients

**DOI:** 10.1186/s12872-025-05015-w

**Published:** 2025-08-28

**Authors:** Gamela Nasr, Aliaa Tarek Mahfouz, Mostafa Wanees Ahmed El husseny, Omnia Kamel, Gamal Shaban, Ramadan Ghaleb

**Affiliations:** 1 Department of Cardiology, Faculty of medicine, Suez Canal university, Ismailia, Egypt; 2 Department of Cardiology, Faculty of medicine, Aswan university, Aswan, Egypt; 3 Department of Adult Cardiology, Aswan Heart Centre, Aswan, Egypt; 4 Department of Cardiology, Faculty of medicine, Fayoum university, Fayoum, Egypt; 5 Department of Cardiology, National heart institute, Cairo, Egypt

**Keywords:** Cardiac resynchronization therapy, QRS duration, Heart failure, Biventricular pacing, Cardiomyopathy, Mechanical remodeling

## Abstract

**Background:**

Cardiac resynchronization therapy (CRT) has been established as a key component in the management of patients with heart failure (HF) with reduced ejection fraction in addition to pharmacologic therapy. Several automatic algorithms have been developed to optimize the timing cycle settings in CRT, especially AV delay which was associated with improvement of the response to CRT. The present study aims to investigate whether the novel device-based SyncAV algorithm could elicit better synchrony and acute hemodynamic response.

**Methods:**

Thirty-six patients (age 52.81 ± 14.45 years; 69% male; 27% with ischemic cardiomyopathy; LV ejection fraction 24.81 ± 5.87%; QRS duration173.64 ± 15.99 ms) with intact atrioventricular conduction (PR interval 184.69 ± 15.43, range 150–210 ms), left bundle branch block, undergoing CRT implantation were prospectively studied. The device was programmed to two biventricular (BiV) pacing modes after the procedure: Nominal AV delay (140/110 ms) optimization (Group A), and SyncAV algorithm with optimized offset minimizing QRS duration (QRSd) (Group B). After each setting, clinical electrocardiographic, and echocardiographic data were collected at 1- and 6-months post implantation.

**Results:**

The intrinsic QRSd (171.50 ± 16.32 ms) was reduced to161.17 ± 19.89 ms after 1 month and to 157.44 ± 24.51ms after 6 months among group A, However, intrinsic QRSd ( 175.78 ± 15.82 ms ) was reduced to 154.00 ± 14.62 ms after 1 month and to 141.00 ± 16.45 ms after 6 months among group B [*P* < 0.001 versus nominal mode]) with significant improvement in clinical and echo criteria and without QRSd prolongation in any patient.

**Conclusion:**

Post-implant electrical optimization in already well-selected patients with left bundle branch block and optimized LV lead position is facilitated by patient-tailored BiV pacing adjusted to intrinsic atrioventricular timing using an automatic device– based algorithm.

## Background

Cardiac resynchronization therapy (CRT) has been established as a key component in management of patients with heart failure (HF) with reduced ejection fraction in addition to pharmacologic therapy and other device-based therapies as it is recommended by the latest heart failure guidelines [1].

CRT has demonstrated in multiple outcome trials its ability to reduce the risk of heart failure and mortality in patients with impaired left ventricular (LV) function and a widened QRS complex [2]. Additionally, CRT has been associated with significant improvements in echocardiographic parameters, including end-diastolic and end-systolic volumes., ejection fraction, right ventricular (RV) function, and left atrial (LA) size [3].

Optimizing both atrioventricular (AV) and ventricular-ventricular (VV) intervals enhances the therapeutic benefits of CRT [4]. Optimizing AV delay through fusion of intrinsic RV depolarization and biventricular pacing can shorten QRS duration (QRSd), which reflects LV activation time. This shortening is linked to LV reverse remodeling, improved response to CRT, and reductions in cardiovascular hospitalizations and mortality.

The optimal AV interval is achieved by synchronizing ventricular systolic and diastolic phases. Common methods for determining the best AV interval include repeated echocardiographic assessments with iterative program adjustments, measuring the velocity time integral (VTI) through Doppler flow and analyzing the timing of two key waveforms, the E (early diastolic) and A (atrial contraction) waves from the LV inflow pattern [5].

However, when the AV delay (AVD) is manually set during rest, it stays constant and doesn’t adjust to changing conditions during physical activity therefore it does not account for variations in intrinsic AV conduction due to physiological or autonomic changes, limiting its effectiveness in different clinical situations.

In 2017, SyncAV algorithm has been introduced, offering the concept of triple wavefront fusion during BiV pacing [6], It measures intrinsic conduction periodically and adjusts AV delays dynamically. This device-based algorithm can be customized for individual patients through specific programming rather than relying on default settings. Studies have shown that the SyncAV algorithm enhances acute electrical synchrony compared to traditional CRT, with further improvements observed through patient-specific optimization of the SyncAV offset [7]. However, it remains unclear whether this individualized BiV pacing, aligned with intrinsic AV timing, leads to mechanical benefits.

## Methods

### Study population

This was a single center, prospective randomized study, that enrolled 36 patients from January 2020 to January 2023, aged 18 or older who had CRT defibrillator or pacemaker implantation after providing written informed consent.

Patients were strictly screened before implant to ensure that all inclusion criteria were met; (a) NYHA classes III-IV; (b) ejection fraction ≤ 35% (either ischemic or non-ischemic cardiomyopathy); (c) sinus rhythm; PR interval < 300 msec, (d) left bundle branch block (LBBB) with a QRS duration of 150 ms or longer (LBBB is defined by a native QRS duration of at least 120 ms, characterized by a broad, notched, or slurred R wave visible in leads I, aVL, V5, and V6. This may include an RS pattern in leads V5 and V6, with no Q waves present in leads I, V5, and V6. Additionally, the R-wave peak time in leads V5 and V6 surpasses 60 ms while remaining within normal parameters in leads V1 through V3), (e) optimal medical therapy for at least 3 months including beta-blockers, mineralocorticoid receptor antagonists (MRAs), Angiotensin Receptor-Neprilysin Inhibitors (ARNI), and sodium-glucose cotransporter-2 inhibitors (SGLT2 inhibitors). It is important to note that SGLT2 inhibitors were either from the start or initiated during follow up. Patient compliance to their medication were ensured during follow up visits. Additionally, blood sugar lowering agents for diabetic patients and anti-ischemic treatment for ischemic patients. Patients were excluded from the study if they had congenital heart diseases, valve repair or replacement surgeries, atrial tachyarrhythmias or frequent atrial or ventricular ectopy, any degree of AV block coronary artery bypass surgery or PCI in the previous 6 weeks. Before device implantation, all subjects underwent 12-lead electro- cardiogram (ECG) and transthoracic echocardiogram.

### Standard trans-thoracic two-dimensional echocardiographic examination

An experienced cardiologist conducted examinations on all patients in the left lateral decubitus position using a General Electric Vingmed Vivid 9 ultrasound system equipped with an M4S transducer.

Standard two-dimensional and M-mode echocardiographic images were acquired from the apical four-chamber, apical two-chamber, and left parasternal views, following the guidelines established by the American Society of Echocardiography to measure LV end-systolic and end-diastolic dimensions in the left parasternal short axis view at the level of papillary muscles, Calculate left ventricular ejection fraction using Teichholz Method, mitral and tricuspid regurgitation was semi-quantitatively graded according to the jet area method.

ECHO parameters on presentation, after implantation and programming, 4-week post implantation and after 6 months follow up were collected and recorded.

### CRT implantation

All patients in this study underwent CRT device implantation following the ESC/EHRA guidelines. All procedures were performed under general anesthesia with axillary venous access. Prior to implantation it was crucial to evaluate the location and extent of myocardial scar tissue by the help of LGE CMRI to avoid placing of the lead in these areas that may result in ineffective CRT response. Fluoroscopic Venography in a 30° left anterior oblique view was done in each procedure to visualize the CS with all its tributaries to estimate the target accessibility based on our experience, available tools and pure anatomical approach. Our first goal was optimum positioning of a Quadripolar LV lead (Quartet 1458Q; Abbott) in lateral or the posterolateral branches in most of the cases to ensure best mechanical synchronization, however, in cases of suboptimal anatomy, it was positioned in anterior or inferior vein. The RV lead (Tendril STS 2088TC; Abbott) or defibrillation electrode (Durata 7122; Abbott) was placed at the right ventricular apex (RVA), while the right atrial (RA) lead (IsoFlex Optim 1944; Abbott) was positioned in the right atrial appendage. Once satisfactory sensing, pacing, and impedance parameters were confirmed, all leads were secured and connected to the pulse generator (Quadra Allure 3242 for CRT-P and Quadra Assura 3371-40 for CRT-D; Abbott).

### CRT programming modes

After CRT implantation, patients were randomly assigned (1:1) into two groups (A&B) with prespecified settings, each of which was programmed for a minimum of 60 s before electrocardiographic data collection. Group (A) Nominal settings algorithm: 18 patients undergoing CRT with optimizing AVD with the usual setting parameters most physicians do; Paced/sensed AVD was optimized as nominal settings as BiV (140/110). Group (B) employed the SyncAV algorithm with personalized offset values for each patient to achieve the narrowest QRS duration (QRSd). For the other 18 patients, the algorithm was set to BiV + SyncAV mode with offset values of 10, 20, 30, 40, or 60 ms, facilitating variable triple-wavefront fusion and QRS morphologies. The offset value producing the narrowest QRSd was considered optimal. Every 256 cycles, the algorithm automatically extended the atrioventricular delay (AVD) to measure the intrinsic AV interval. In the subsequent 256 cycles, the AVD was modified using the default SyncAV offset, calculated as 50 ms subtracted from the measured intrinsic AV interval. The VV (LV-RV) interval was consistently set between 20 and 30 ms, a common practice in our CRT programming, as LV prepacing is typically favored for CRT timing cycles.

### Follow-up

The enrolled patients had been followed up at 30- and 180-days post procedure. Heart failure symptoms, major adverse cardiovascular events (MACE) at any time were reported including sudden cardiac death, and heart failure hospitalization. Clinical examination, 6MWT, twelve lead ECG (mainly QRSd) and follow up echocardiography were performed and compared to the baseline studies done before CRT implantation.

### Statistical methods

All statistical analyses were performed using SPSS software (Statistical Package for the Social Sciences; SPSS Inc., Chicago, IL, USA), version 22. Quantitative data were presented as mean ± standard deviation (SD) and median (range), while qualitative data were expressed as frequencies (number of cases) and percentages (relative frequencies), where applicable. The Student’s t-test was applied for comparing normally distributed quantitative variables, whereas the Mann-Whitney U test and Kruskal-Wallis test were used for non-normally distributed data. Repeated measures ANOVA was utilized to analyze repeated echocardiographic measurements within groups over time and to evaluate group-time interactions. Categorical data were compared using the Chi-square (χ²) test, with the Fisher’s Exact test applied when expected frequencies were below five. The Friedman test was used to examine changes in qualitative data over time. Correlations between variables were assessed using Pearson’s correlation test. A two-tailed p-value of less than 0.05 was considered statistically significant.

## Results

### Baseline characteristics

Mean age of the cohort was 52.8 ± 14.4 years and 25 (69%) were males. Ischemic cardiomyopathy was the underlying cardiac disease in 10 (27.8%) patients, while 26 patients (72.2%) suffered of dilated cardiomyopathy. Among DCM patients, 14 patients of them (77.8%) were in group A, while group B was observed to suffer from ICM with greater percentage than those in group A (33.3% Vs 22.2% respectively) yet this was statistically non-significant. Group B were younger by a non-statistically significant difference. Group B had tendency towards less weight and better BMI values compared with group A with *p* = 0.021 and 0.005 respectively.

At implant, 25 patients (69.4%) were at NYHA Class III and 11 patients (30.6%) at NYHA Class IV. There was no significant difference between both groups in the baseline clinical parameters data including mean 6MWT, and NYHA class. The mean LVEF at implant was 24.8% ± 5.87%. Heart rate at rest was 87.14 ± 13.9 (range 63–138 ms) with an intrinsic QRS of 173.64 ± 15.9 ms (range 150–200 ms) There was no significant difference between both groups in Echo data including LVEF, LVESD & degree of MR (Table 2) as well as average HR and QRS duration. The average baseline PR interval was 184 ± 15.4 milliseconds, with only 1 out of 18 patients in Group B exceeding 200 milliseconds, showing a statistically significant difference in this group (Tables 1 and 2).

**Table 1:** Demographic Data of all patients included in this study (*n* = 36)

Variable name	Total (*n*=36)		Group A (*n*=18)		Group B (*n*=18)		*P* value
Age (years)							0.848
•Mean ± SD	52.81 ± 14.45		53.28 ± 11.31		52.33 ± 17.36		
Gender, *n* (%)							0.070
•Male	25	(69.4)	10	(55.6)	15	(83.3)	
•Female	11	(30.6)	8	(44.4)	3	(16.7)	
Weight (kg)							**0.021**
•Mean ± SD	82.01 ± 16.59		88.31 ± 14.80		87.57 ± 16.25		
Height (cm)							0.269
•Mean ± SD	165.43 ± 9.59		163.64 ± 10.40		167.22 ± 8.63		
BMI (kg/m^2^)							**0.005**
•Mean ± SD	30.17 ± 6.79		33.23 ± 6.44		27.10 ± 5.79		
• Under weight (<18.5)	2	(5.6)	0	(0.0)	2	(11.1)	0.077
•Normal (18.5 - 24.9)	5	(13.9)	1	(5.6)	4	(22.2)	
•Overweight (25 - 29.9)	10	(27.8)	4	(22.2)	6	(33.3)	
•Obese ≥ 30	19	(52.8)	13	(72.2)	6	(33.3)	
Comorbidities, *n* (%)							
•DM	10	(27.8)	5	(27.8)	5	(27.8)	1
•HTN	14	(38.9)	7	(38.9)	7	(38.9)	1
Type of cardiomyopathy, *n* (%)							0.457
•DCM	26	(72.2)	14	(77.8)	12	(66.7)	
•ICM	10	(27.8)	4	(22.2)	6	(33.3)	

Quantitative data are presented as mean ± SD and median (range), qualitative data are presented as number (percentage). Significance defined by *p* < 0.05

*6MWT* Six-minute walking test, *BMI* Body mass index, *DCM* Dilated Cardiomyopathy, *DM* Diabetes mellites, *ICM* Ischemic cardiomyopathy, *HTN* Hypertension

Bold values defined by *p* < 0.05

**Table 2:** Baseline clinical parameters, ECG findings, echocardiographic findings of the studied participants (Pre-implantation)

Variable name	Total (*n*=36)		Group A (*n*=18)		Group B (*n*=18)		*P* value
6MWT (m)							0.697
•Mean ± SD	199.88 ± 93.03		206.63 ± 93.75		193.89 ± 94.68		
NYHA class, *n* (%)							0.278
•III	25	(69.4)	11	(61.1)	14	(77.8)	
•IV	11	(30.6)	7	(38.9)	4	(22.2)	
ECG Findings:							
HR (beats/minute)							0.203
•Mean ± SD	87.14 ± 13.93		90.28 ± 16.25		84.00 ± 10.70		
PR interval (ms)							**0.050**
•Mean ± SD	184.69 ± 15.43		179.47 ± 15.87		189.61 ± 13.66		
QRSd (ms)							0.430
•Mean ± SD	173.64 ± 15.99		171.50 ± 16.32		175.78 ± 15.82		
Echocardiographic Findings:							
LVESD (mm)							0.702
•Mean ± SD	6.25 ± 0.85		6.19 ± 0.77		6.31 ± 0.95		
LV EF (%)							0.889
•Mean ± SD	24.81 ± 5.87		24.94 ± 5.87		24.67 ± 6.04		
MR grade							0.362
•I	11	(31.4)	5	(29.4)	6	(33.3)	
•II	7	(20.0)	3	(17.6)	4	(22.2)	
•III	12	(34.3)	8	(47.1)	4	(22.2)	
•IV	5	(14.3)	1	(5.9)	4	(22.2)	

Quantitative data are presented as mean ± SD and median (range), qualitative data are presented as number (percentage). Significance defined by *p* < 0.05

*6MWT* Six-minute walking test, *ECG* Electrocardiography, *LVEF* Left ventricle ejection fraction, *LVESD* Left ventricle end-systolic diameter, *MR* Mitral regurge, *DM* Diabetes mellites, *ICM* Ischemic cardiomyopathy, *QRSd* QRS duration, *NYHA* New York Heart Association

Bold values defined by *p* < 0.05

### CRT implantation

Using a coronary sinus venogram in a 30° left anterior oblique fluoroscopic view, a quadripolar multipoint pacing LV lead (36 leads; 100%) were successfully implanted in the posterolateral vein branch of the free wall of the left ventricle in 26 cases (72.2%). These leads were evenly distributed across both groups, with 14 cases (77.8%) in group A. In three cases (8.3%), anatomical variations of the cardiac veins necessitated implantation at anterior or anterolateral sites. The distribution of the remaining implantation sites is detailed in (Table 3). BiV pacing was checked immediately after implantation and every visit post to ensure that the programmed parameters with the AV delay mode providing the best BiV pacing percentage. The total cohort had BiV pacing ranged between 97 and 99%. During follow-up period (6 months), no MACEs (sudden cardiac death, or heart failure hospitalization) were detected in both groups.

**Table 3:** Site of LV lead position among cases of both groups

Variable name	Total (n=36)		Group A (n=18)		Group B (*n*=18)		*P* value
LV lead position							0.464
•PL branch	26	(72.2)	14	(77.8)	12	(66.7)	
•Anterior	3	(8.3)	2	(11.1)	1	(5.6)	
•Lateral	4	(11.1)	2	(11.1)	2	(11.1)	
•Posterior	3	(8.3)	0	(0.0)	3	(16.7)	

Quantitative data are presented as mean ± SD and median (range), qualitative data are presented as number (percentage). Significance defined by *p* < 0.05

*PL* posterolateral branch

### Electrical, mechanical and clinical synchronization difference between the two groups

**Fig. 1:**
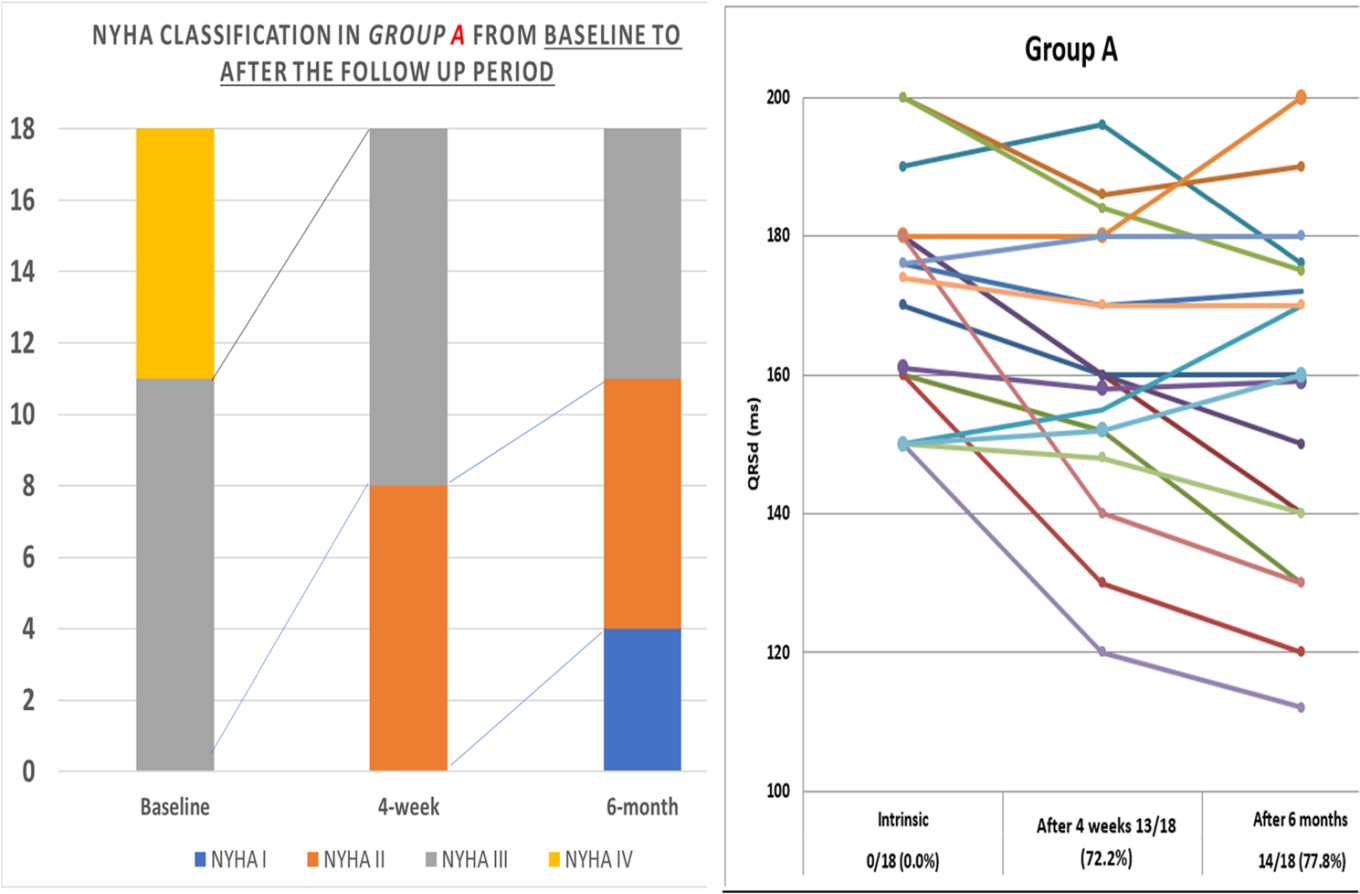
Side-by-side panel showing NYHA classification in patient in Group A from baseline to after the follow up period (left) and distribution of QRS duration (QRSd) among 18 patients of group A while using the nominal AV delay algorithm; individual comparison between basic QRSd before implantation and after 4 week and 6 months post CRT implantation (right). NYHA: New York Heart Association, QRSd: QRS duration

Either after four weeks or after six months of CRT implantation; most of the patients in group A had improved significantly from the initial values regarding clinical parameters (NYHA class and 6MWT) with *P* < 0.001. Regarding QRS width of group A didn’t decrease significantly at four weeks from initial value, but at 6 months it decreased by weak significance (157.44 ± 24.51 msec, *p* = 0.02). Additionally, LVEF and LVESD didn’t show any significant improvement in this group either in short or long-term follow-up (Table 4), (Figs. 1 and 3).

**Table 4:** Clinical parameters, QRSd and Echocardiographic finding within group A and B from baseline to after the follow up period

*Variables (Mean ± SD)*	*Group A*			*Group B*		
	*Baseline*	*4-week*	*6-month*	*Baseline*	*4-week*	*6-month*
*6MWT (m)*	206.63 ± 93.75	262.78 ± 76.14	302.22 ± 99.09	193.89 ± 94.68	282.59 ± 124.56	369.22 ± 132.57
	P value **<0.001**			P value **0.001**		
*QRSd (ms)*	171.50 ± 16.32	161.17 ± 19.89	157.44 ± 24.51	175.78 ± 15.82	154.00 ± 14.62	141.00 ± 16.45
	P value **0.020**			P value **<0.001**		
*LVEF (%)*	24.94 ± 5.87	24.17 ± 5.28	27.17 ± 7.49	24.67 ± 6.04	25.47 ± 6.89	31.33 ± 7.52
	P value 0.062			**P value <0.001**		
*LVESD (mm)*	6.19 ± 0.77	6.14 ± 0.79	5.85 ± 1.12	6.31 ± 0.95	5.98 ± 0.99	5.35 ± 0.87
	P value 0.327			P value **<0.001**		

Quantitative data are presented as mean ± SD and median (range), qualitative data are presented as number (percentage). Significance defined by *p* < 0.05

*6MWT* Six-minute walking test, *LVEF* Left ventricle ejection fraction, *LVESD* Left ventricle end-systolic diameter, *QRSd* QRS duration

Bold values defined by *p* < 0.05

**Fig. 2:**
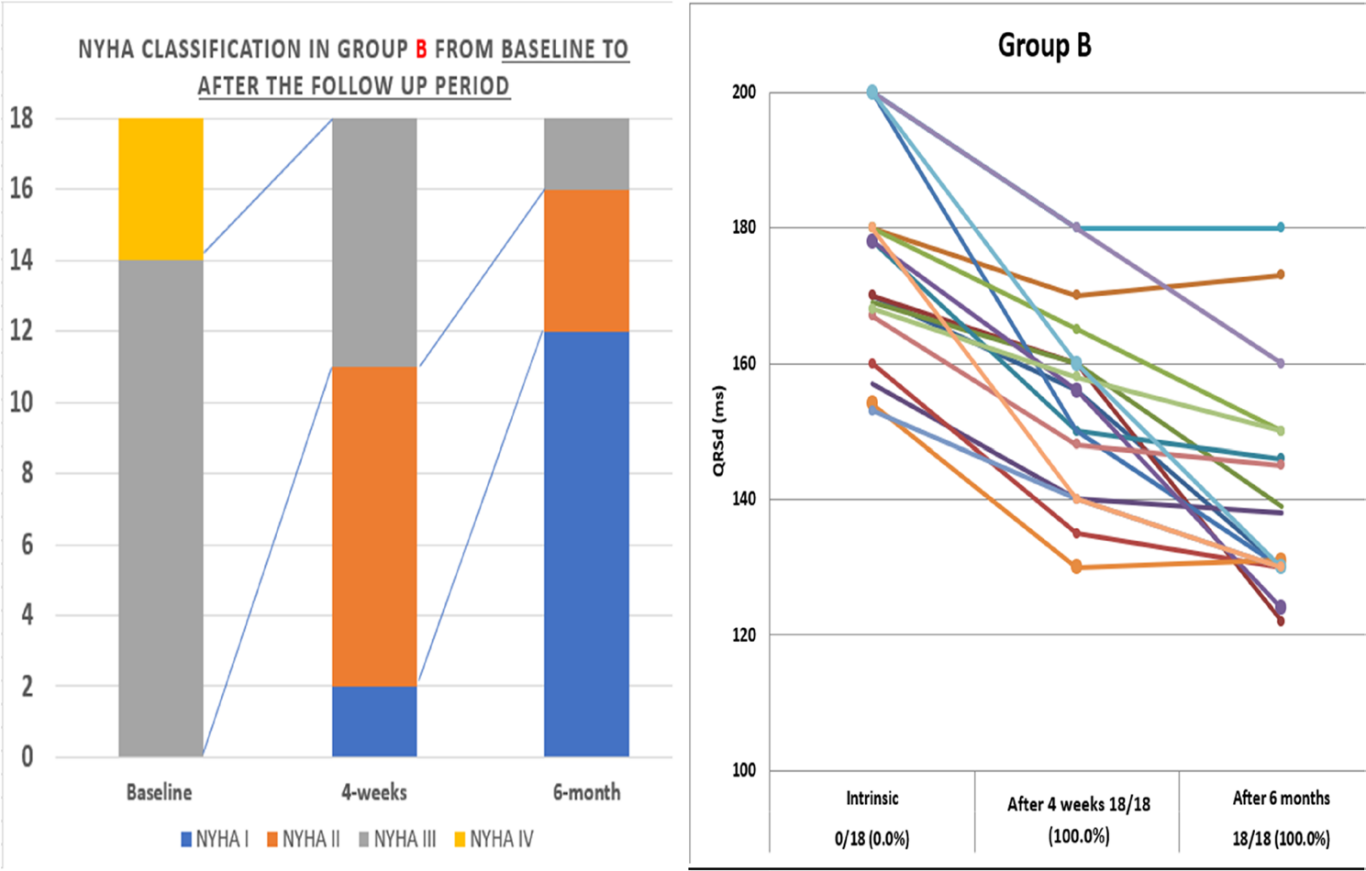
Side-by-side panel showing NYHA classification in patient in Group B from baseline to after the follow up period (left) and distribution of QRS duration (QRSD) among 18 patients of group B while using the Sync AV delay algorithm; individual comparison showing the highly significant reduction among all patients after the short and long term follow up relative to the baseline QRSd (right). NYHA: New York Heart Association, QRSd: QRS duration

**Fig. 3:**
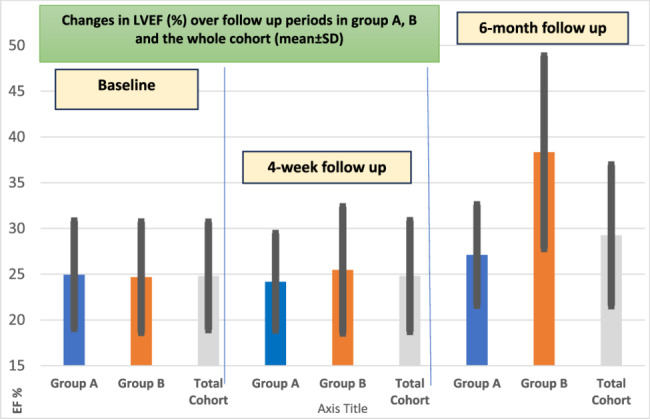
The changes of LV ejection fraction over the study follow-up period in group A, group B and the total cohort (mean** ±**SD) with statistically significant improvement of LVEF in group B in 4-weeks and 6-month follow-up

**Fig. 4:**
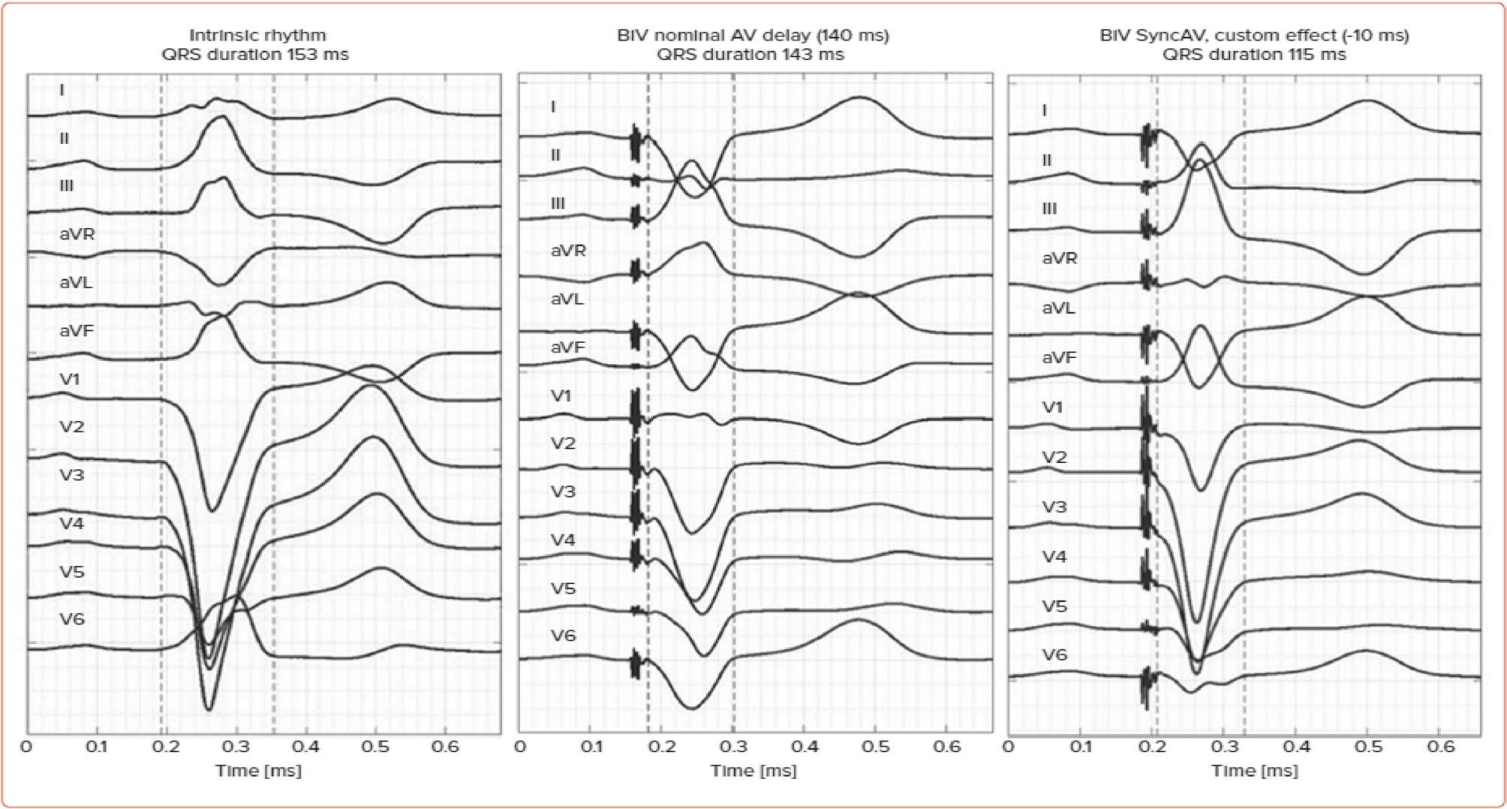
Twelve lead ECG with 50 mm speed QRSd comparison between nominal AV delay and syncAV for case 5 in group B; (A) Intrinsic rhythm with QRS width 153ms, (B) QRSd: 143ms with BiV nominal AVD, (C) QRSd: 115ms with syncAV (with -10ms offset)

**Fig. 5:**
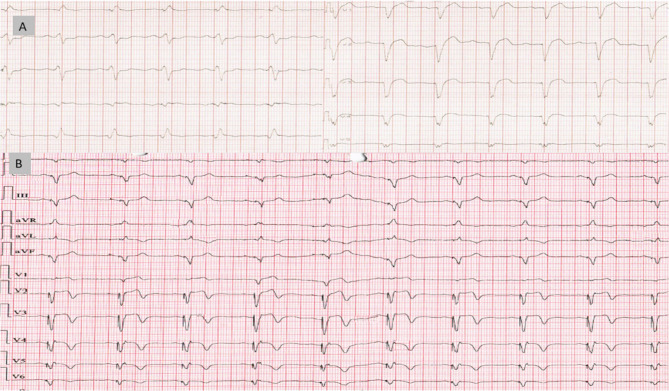
Twelve lead ECG with 25 mm speed QRSd comparison between intrinsic QRSd and syncAV for case 8 in group B; (A) Intrinsic rhythm with QRS width 160ms, (B) QRSd: 125 ms with syncAV (with -20ms offset)

**Fig. 6:**
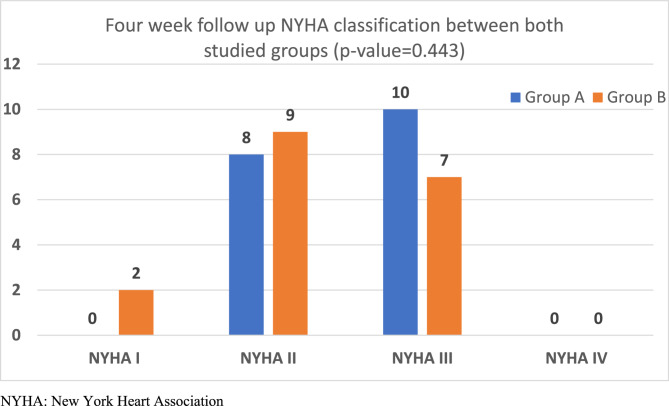
NYHA classification in both *Groups *at 4-week follow-up

**Fig. 7:**
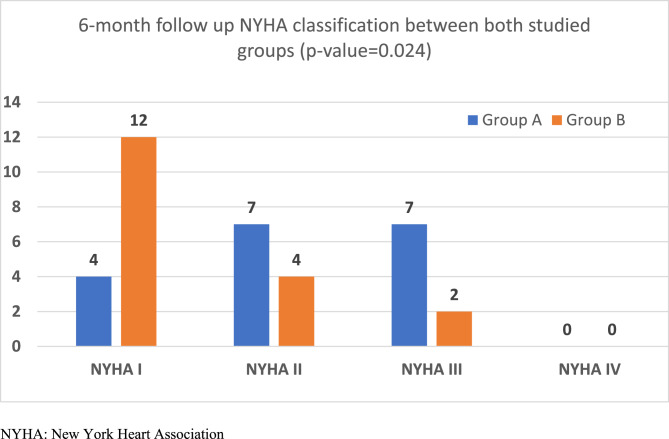
NYHA classification in both *Groups *at 6-month follow-up

While in Group B with Sync AV delay optimization algorithm, the patients had significant improvement in clinical, echocardiographic and QRSd either at one or six months of follow up (Figs. 2 and 3). The mean QRSd had significantly reduced in group B either after four weeks or after 6 months compared to the baseline QRSd; (154.00 ± 14.62 Vs 17141.00 ± 16.45 ms) were the mean durations after four week and 6 months respectively coming from 5.78 ± 15.82 ms as the baseline mean duration; P < 0.001 (Figs. 2, 4 and 5). This notable reduction in QRS duration corresponded with a significant enhancement in NYHA classification and 6MWT performance. By the end of the study, no patients came complaining of NYHA IV, only two patients remained in NYHA class III, compared to 14 at the study’s outset, while 12 patients (66.7%) had improved to NYHA class I after six months (Figs. 2, 6 and 7). Furthermore, echocardiographic parameters, including mean LVEF and LVESV, showed significant improvement in group B over the six months (LVEF increased from 24.67 ± 6.04% to 31.33 ± 7.52%, *p* < 0.001; LVESD decreased from 6.31 ± 0.95 mm to 5.35 ± 0.87 mm, *p* < 0.001). (Table 4) (Fig. 3). Our study results didn’t show any statistically significant privilege regards one SYNC AV offset over the others’ however − 40ms offset happened to be the most used one to achieve the narrowest QRS width among group B patients.

#### Inter-group comparisons

A four-week follow-up resulted in better mechanical synchrony and acute clinical improvement among group B regarding NYHA class and 6 MWT, yet with no statistical significance. QRS narrowing was of no significance after four weeks in both groups. There were no significant changes in LVEF and LVESD between the two groups after four week follow up (Table 5) (Figs. 3 and 7).

**Table 5:** Inter-group comparisonsof clinical, electrocardiographic, echocardiographic parameters data between both studied groups* at four week and six Months follow-up*

*Variables (Mean ± SD)*	*4-week follow up*			*6-month follow-up*		
	*Group A*	*Group B*	*All cohort*	*Group A*	*Group B*	*All cohort*
*6MWT (m)*	262.78 ± 76.14	282.59 ±124.56	272.40 ± 101.49	302.22 ± 99.09	369.22 ± 132.57	335.72 ± 120.25
	P value 0.05			P value 0.095		
*QRSd (ms)*	161.17 ± 19.89	154.00 ± 14.62	157.69 ± 17.65	157.44 ± 24.51	141.00 ± 16.45	149.22 ± 22.19
	P value 0.235			***P* value 0.024**		
*LVEF (%)*	24.17 ± 5.28	25.47 ± 6.89	24.80 ± 6.06	27.17 ± 7.49	31.33 ± 7.52	29.25 ± 7.69
	P value 0.533			P **value 0.03**		
*LVESD (mm)*	6.14 ± 0.79	5.98 ± 0.99	6.06 ± 0.88	5.85 ± 1.12	5.35 ± 0.87	5.59 ± 1.02
	P value 0.901			P value 0.273		

Quantitative data are presented as mean ± SD and median (range), qualitative data are presented as number (percentage). Significance defined by *p* < 0.05

*6MWT* Six-minute walking test, *LVEF* Left ventricle ejection fraction, *LVESD* Left ventricle end-systolic diameter, *QRSd* QRS duration

Bold values defined by *p* < 0.05

At 6 months follow-up,QRS width showed statistically significant improvement among group B with mean QRS141.00 ± 16.45 ms, in comparison to group A that decreased to 157.44 ± 24.51 ms; with *P* = 0.024. Mean Values of LV systolic function and LVESD also improved in group B with better values than group A after 6 months with statistically significant difference for LV systolic function results (Table 5) (Figs. 2 and 7). NYHA classification of group B improved in comparison to group A, with 77.8% from all study patients who came suffering from NYHA III were from group A. However, this was statistically not significant with *P* = 0.024. Similarly, with a statistical insignificance, meters of 6 MW test were longer in group B than group A (369.22 ± 132.57 Vs 302.22 ± 99.09 m) respectively, *P* = 0.095.

Quantitative data are presented as mean ± SD and median (range), qualitative data are presented as number (percentage). Significance defined by *p* < 0.05. 6MWT: Six-minute walking test, BMI: Body mass index, DCM: Dilated Cardiomyopathy, DM: Diabetes mellites, ICM: Ischemic cardiomyopathy, HTN: Hypertension.

## Discussion

The 2000 s have been described as the “device era” [8], with CRT revolutionizing the management of patients with impaired LV function and wide QRS complexes. Landmark trials confirmed that CRT should be regarded as an additional resource to pharmacological therapy, rather than a sequential therapy initiated after pharmacological treatments [9].

The RAFT study emphasized that any degree of QRS narrowing was associated with a greater reduction in mortality and heart failure, irrespective of the baseline QRS morphology. Improvement of electrical synchrony could be achieved by narrowing of the QRS (defined as a reduction of QRS duration [QRSd] of at least 20 ms or > 20% compared to baseline QRS) is associated with structural reverse remodeling in CRT recipients and electrical as well as mechanical synchrony are both predictors for long-term outcome in CRT [10].

Suboptimal AVD is a key contributor to CRT nonresponse. Recently, device-based algorithms (DBAs) have been developed to optimize AVD using intracardiac electrograms, but their long-term efficacy remains uncertain. NICE-CRT trial highlighted the potential of dynamic AV delay programming to enhance resynchronization by targeting fusion with intrinsic conduction, offering greater benefits than simultaneous biventricular pacing. Therefore, individualized programming of SyncAV with a tailored offset appears to be a worthwhile approach [6]. These results came along with our study results that showed significant improvement of QRS duration especially at the 6 months follow up in parallel with clinical improvement.

The findings of our study clearly showed that post-CRT optimization using the SyncAV algorithm was more effective than nominal AV settings in addressing both electrical and mechanical asynchrony in carefully selected patients with LBBB. Conventional simultaneous BiV pacing with short or nominal AVD neglects intrinsic RV activation, which may reduce CRT efficacy due to the absence of physiological intrinsic conduction.

By contrast, optimizing device settings through wavefront fusion can enhance the response rate to CRT. The SyncAV algorithm times RV pacing to coincide with the initiation of intrinsic conduction, which depolarizes parts of the right ventricle and septum, while the preactivated LV pacing wavefront merges with both the RV pacing wavefront and the intrinsic wavefront. Ter Horst et al. highlighted that triple wavefront fusion leads to the most significant hemodynamic improvements in the majority of CRT patients [11].

Based on the employed criteria of the clinical response definition drowned upon the four pivotal CRT studies (MIRACLE, MIRACLE ICD, CONTAK CD, and MUSTIC SR); our total study population showed improvement in clinical, echocardiographic and QRS duration but without significant difference between the two groups. Interestingly individualized AVD delay programming using Sync AV delay algorithm achieved reduction of QRS width in 100% of group B patients after the short term follow up and that was coincided with weak significant improvement in NYHA class and 6MWT among those patients as was evidenced by Wang et al. who used aVTI as an indicator of acute hemodynamic improvement of SyncAV + BiV pacing. The modes produced the highest aVTI scores, aligning with findings by Wang et al., who reported that QRSd shortening is strongly associated with acute enhancements in hemodynamic parameters [6].

However, after 6 months, our data revealed significant improvement of all clinical and ECHO data among group B proving the effect of individualized optimization of AV delay by the Sync AV algorithm that was significantly better than nominal AV optimization. Importantly, the 6-month follow-up in this study revealed that SyncAV contributed to better LV remodeling, with assocaited reduced LVESD, increased LVEF, and notable improvements in the NYHA class that was manifested by the evidence that 66.7% of group B patients was included in NYHA I class and the, 6MWT was significantly increased especially in comparison to the fixed nominal mode, all these came along with significant response in echo paraments.

Notably, Sync AV delay optimization with individualized offset between (−20 and − 60 ms) achieved very strong significant QRS width narrowing among the whole 18 patients that were included in this group, this electrical synchronization aligned with the mechanical and hemodynamic improvements observed in SyncAV CRT, enabling effective post-implant optimization in typical clinical settings. These findings are consistent with the NICE CRT trial [12], which confirmed that dynamic AV delay programming, aimed at enhancing fusion with intrinsic conduction, achieves greater resynchronization than simultaneous biventricular pacing.

Limitations: Working on our study, we had some limitations worth to be mentioned. In the first place, the sample size is relatively small due to the limited number of patients implanted with CRT devices with SyncAV algorithm in our center and because of the covid 19 circumstances back then that was an obstacle of the whole medical services especially the non-urgent ones. The outcomes were reported at 1–6 months; a more long follow up period could yield more results in future studies. Females were under-represented in the study group (30%), as generally females less frequently present to medical services in rural areas in developing countries. The optimal lead position was determined using electrical parameters rather than echocardiographic assessment based on regions of latest contraction, a method associated with improved outcomes compared to traditional placement but limited by issues of accuracy, reproducibility, extended procedural times, and increased radiation exposure. Our study exclusively included patients with LBBB. It remains uncertain whether individuals with non-LBBB patterns could also benefit from such post-implant optimization. Another point to be improved in future studies, is the use of a more objective echocardiographic hemodynamic parameters for assessment, as the left Ventricular Outflow Tract Velocity-Time Integral (LVOT-VTI), ejection time and synchronization indices such as septal-to-posterior wall motion delay (SPWMD). In our study, cardiac MRI (CMR) was utilized for volume and function assessment during follow-up, yet not all cases due to COVID pandemic and claustrophobia in some patients, thus it was not added to our final statistical analysis.

 Finally, the long-term impact of CRT programming for electrical resynchronization, combined with a dynamic device-based platform, is being prospectively evaluated in ongoing randomized trials and warrants further investigation [13].

## Conclusion

Post-implant electrical optimization in already well-selected patients with left bundle branch block and optimized LV lead position is facilitated by patient-tailored BiV pacing adjusted to intrinsic atrioventricular timing using an automatic device– based algorithm.

## Data Availability

The datasets used and/or analyzed during the current study are available from the corresponding author on reasonable request. Availability of data and materialsThe original contributions presented in the study can be inquired directly from the corresponding author.

## Abbreviations

6MWT: Six-minute walking test
AV: Atrioventricular
AVD: Atrio-Ventricular delay
BiV: Biventricular
BMI: Body mass index
CRT: Cardiac resynchronization therapy
DCM: Dilated Cardiomyopathy
HF: Heart Failure
HR: Heart rate
ICD: Implantable Cardioverter Defibrillator
ICM: Ischemic cardiomyopathy
LA: Left atrial
LV: Left ventricular
LVEF: Left ventricle ejection fraction
LVESD: Left ventricle end-systolic diameter
LVADs: Left Ventricular Assist Devices
MACE: Major adverse cardiovascular events
MR: Mitral regurge
NYHA: New York Heart Association
QRSd: QRS duration
RV: Right ventricular
VAS: Ventricular Arrhythmias
VT: Ventricular Tachycardia
VTI: Velocity time integral
VV: Ventricular-ventricular
